# Low-Intensity Exercise Modulates Gut Microbiota to Fight Against Radiation-Induced Gut Toxicity in Mouse Models

**DOI:** 10.3389/fcell.2021.706755

**Published:** 2021-10-21

**Authors:** Bin Wang, Yu-xiao Jin, Jia-li Dong, Hui-wen Xiao, Shu-qin Zhang, Yuan Li, Zhi-yuan Chen, Xiao-dong Yang, Sai-jun Fan, Ming Cui

**Affiliations:** ^1^Tianjin Key Laboratory of Radiation Medicine and Molecular Nuclear Medicine, Institute of Radiation Medicine, Chinese Academy of Medical Sciences and Peking Union Medical College, Tianjin, China; ^2^Department of General Surgery, The Second Affiliated Hospital of Soochow University, Suzhou, China; ^3^Department of Anesthesiology, Changshu No. 2 People’s Hospital, Changshu, China

**Keywords:** radiotherapy, radiation-induced gastrointestinal tract toxicity, intestinal inflammation, low-intensity exercise, walking, gut microbiota, *Akkermansia muciniphila*

## Abstract

Radiation-induced gastrointestinal (GI) tract toxicity halts radiotherapy and degrades the prognosis of cancer patients. Physical activity defined as “any bodily movement produced by skeletal muscle that requires energy expenditure” is a beneficial lifestyle modification for health. Here, we investigate whether walking, a low-intensity form of exercise, could alleviate intestinal radiation injury. Short-term (15 days) walking protected against radiation-induced GI tract toxicity in both male and female mice, as judged by longer colons, denser intestinal villi, more goblet cells, and lower expression of inflammation-related genes in the small intestines. High-throughput sequencing and untargeted metabolomics analysis showed that walking restructured the gut microbiota configuration, such as elevated *Akkermansia muciniphila*, and reprogramed the gut metabolome of irradiated mice. Deletion of gut flora erased the radioprotection of walking, and the abdomen local irradiated recipients who received fecal microbiome from donors with walking treatment exhibited milder intestinal toxicity. Oral gavage of *A. muciniphila* mitigated the radiation-induced GI tract injury. Importantly, walking did not change the tumor growth after radiotherapy. Together, our findings provide novel insights into walking and underpin that walking is a safe and effective form to protect against GI syndrome of patients with radiotherapy without financial burden in a preclinical setting.

## Introduction

As non-infectious diseases, cancers have been attributed as the leading cause of death globally. It is estimated that there will be at least 10,000,000 new cases of cancer and 6,800,000 cancer deaths from 2018 to 2040^1^. Radiation therapy serves about 4,70,000 patients per year in the United States, and up to 50% of cancer patients will undergo radiotherapy for either curative or adjuvant purposes (Citrin, 2017). Radiotherapy is the first-line treatment for multiple cancers, including head and neck tumors and abdominopelvic tumors. Although ionizing radiation kills malignant tumor cells effectively, early and late adverse side effects are common and grievously intertwined with the remedy (De Ruysscher et al., 2019; Romesser et al., 2019). Iatrogenic local irradiation for abdominopelvic cancers aligns with varying degrees of gastrointestinal (GI) tract complications, covering nausea, malabsorption, diarrhea, and intestinal obstruction, which halt radiotherapy prematurely and degrade the life quality of patients (Hauer-Jensen et al., 2014). To date, safe and effective therapeutic options have been scarce to fight against these complications in clinical application (Vozenin et al., 2019; Whelan et al., 2019).

Bad living habits, such as smoking, drinking, and sedentary lifestyle, emerge as potential pitfalls propelling nearly half of cancers (Islami et al., 2018). The sedentariness has been proved to precipitate the occurrence and development of several cancers (Kerr et al., 2017). Lately, mounting evidence corroborates that physical activity is beneficial for health, including adjusting metabolism, preventing cancer occurrence (Hespanhol Junior et al., 2015; Geng et al., 2019; Rezende et al., 2019; Takahashi et al., 2019), and even synergizing cancer treatment as an immune adjuvant or chemosensitizer (Betof et al., 2015; Febbraio, 2017; Duggal et al., 2019). Heretofore, most researches focus on vigorous exercise, which carries potential cardiovascular risk and is not suitable for frail patients with surgery or radiotherapy (Marijon et al., 2011). As a form of low-intensity exercise, walking has been proved to improve mental health and sleep quality and to prolong the progression-free survival of patients with locally advanced or metastatic colorectal cancer (CRC; Hamer and Steptoe, 2008; Oja et al., 2018; Guercio et al., 2019; Tang et al., 2019). However, whether walking can be employed as a rehabilitation strategy for cancer patients with radiotherapy remains unidentified.

Neonatal gut is colonized by microorganisms immediately following birth. The gut microbiome of infant is modified with development and participates in the formation and improvement of innate and adaptive immunity (Milani et al., 2017). Recently, studies on gut microbiota have experienced a renaissance and explored the tight relationships between enteric flora and host’s health. On the one hand, the gut microbiota modulates metabolism and maintains energy balance of hosts (Lim et al., 2017). On the other hand, intestinal flora dysbiosis precipitates a broad range of intra-intestinal diseases, such as inflammatory bowel disease (IBD) and CRC, and extra-intestinal diseases, covering cardiovascular disorders, diabetes, obesity, and neurodegenerative diseases (Gérard, 2016; Pistollato et al., 2016; Tang et al., 2017; Leiva-Gea et al., 2018; Ostojic, 2018; Fattorusso et al., 2019). Our previous studies have identified the vital roles of intestinal microbes in rehabilitation of radiation-induced GI tract toxicity (Cui et al., 2017). Notwithstanding that heredity, diet, and antibiotic usage are key determinants for gut flora configuration, lifestyle, stress, and exercise have been proven to educate and tune the gut microbiota community as well (Conlon and Bird, 2014; Zhong et al., 2019). Notably, physical exercise modifies the gut bacteria configuration to benefit the host’s haleness (Monda et al., 2017; de Sire et al., 2020). In this study, we reported that short-term walking reshaped the gut microbiota and mitigated radiation-induced GI toxicity in both male and female mice without accelerating the proliferation of cancer cells. Further exploration demonstrated that the radioprotective effects of walking were partly dependent on gut microbiota, such as *Akkermansia muciniphila*. In brief, our findings provide new insights into the function and underlying protective mechanism of walking in the context of intestinal radiation toxicity in a preclinical experimental setting.

## Materials and Methods

### Mice

Male/female 6- to 8-week-old C57BL/6J mice or 4-week-old male BALB/c athymic nude mice were purchased from Beijing Huafukang Bioscience Co., Inc. (Beijing, China). Mice were housed in the specific pathogen-free (SPF) level animal facility at the Institute of Radiation Medicine (IRM), the Chinese Academy of Medical Sciences (CAMS), and maintained in an enriched environment with a temperature-controlled room in a 12-h light–dark cycle, with food and water available. Before the experiment, the mice were adapted to the experimental environment for a week. Animal experiments were performed according to the institutional guidelines approved by the Animal Care and Ethics Committee of IRM-PUMC (the Ethical Approval number is IRM-DWLL-2019096), which complied with the Guide for the Care and Use of Laboratory Animals and the National Institutes of Health Guide for the Care and Use of Laboratory Animals.

### Walking Protocol

The speed of walking (defined as less than 6 m/min in mouse models) was determined firstly according to the previous studies (Bellardita and Kiehn, 2015; Caggiano et al., 2018). Analysis of the movement behavior and the breath measurement of mice showed that compared with 6 m/min, 35 min of 1 m/min walking is a sustainable way of exercise for mice under pathological conditions, which ensures the quality and quantity of the walking (Supplementary Figure 1). Taking the physical exercise ability of the irradiated mice or patients with radiotherapy, 1 m/min walking was finally selected for intervention in the mice. In order to synchronize the segmented radiotherapy strategy for clinical cancer patients, walking strategy was adjusted to 6 days per week. Due to the physical fitness of the BALB/c athymic nude mice, the daily time and speed of walking were reduced on the basis of walking in C57BL/6J mice (30 min of 1 m/min). All mice completed the walking with quality and quantity.

### Radiation Study and Experimental Group

A Gammacell-40 ^137^Cs irradiator (Atomic Energy of Canada Limited, Chalk River, ON, Canada) at a dose rate of 0.88 Gy/min was used for all experiments (Con, control; TAI, total abdominal irradiation; W, walking; FMT, fecal microbiota transplantation; ABX, antibiotic cocktail; ABXW, antibiotic cocktail and walking). The C57BL/6J mice were divided into Con, TAI, TAI + W, TAI + FMT, ABX, and ABXW five groups; and mice in each group except for Con group received 12 Gy of γ-ray TAI when the body weight reached 19–20 g (Li et al., 2020a). The mice in TAI + W group maintained walking on the treadmill (Beijing Zhongshidichuang Science and Technology Development Co., Ltd., Beijing, China) for 15 days (1 m/min, 35 min/day, 6 days/week) following radiation exposure, while TAI group was maintained on fasting and water deprivation for 35 min to eliminate the influence of food and water. For TAI + FMT group, abdominal local irradiated mice were administrated with fecal microbiome via oral route (Cui et al., 2017). The donors were abdominal local irradiated male C57BL/6J mice with walking treatment (based on the “Walking protocol”). The mice in ABX group were housed with drinking water supplemented with an antibiotic cocktail (ABX) (vancomycin, metronidazole, ciprofloxacin, ampicillin, and streptomycin) that can subvert existing gut microbes (Xiao et al., 2020), while ABXW group maintained the walking treatment for 15 days during ABX treatment. During the non-experimental period, the water and food were available *ad libitum*.

### Culture of *Akkermansia muciniphila* and *Akkermansia muciniphila* Transplantation Treatment

*A. muciniphila* MucT (ATCC BAA-835) was cultured in brain heart infusion broth containing 10 mg/L of resazurin (an oxidation–reduction indicator) under strict anaerobic conditions. A representative culture stock was used to determine the CFU/ml under anaerobic conditions by plate counting using mucin media containing 1% agarose. This culture was diluted with anaerobic phosphate-buffered saline (PBS) to a final concentration of 1.5 × 10^8^ CFU/100 μl. To explore the effects of *A. muciniphila* on radiation-induced intestinal injury, the irradiated mice (12 Gy of TAI) were treated with an oral administration of *A. muciniphila* (1.5 × 10^8^ CFU) suspended in sterile PBS for 15 days, while the contrast mice were given sterile PBS with equivalent volume. For the quantitative analysis of *A. muciniphila* in colon, the colons of mice were cut lengthwise; and few feces and mucus layer were scraped off with sterile cotton brush. Then the DNA was extracted by using TIANamp Stool DNA kit (TIANGEN, Beijing, China) and used for q-PCR.

### Cell Culture

Human CRC cell line HCT-8 or human lung cancer cell line A549 were obtained from the American Type Culture Collection (ATCC) and certified to be mycoplasma-free. The cells were cultured with 10% fetal bovine serum (Gibco, Grand Island, NY, United States), 100 U/ml of penicillin, and 100 mg/ml of streptomycin and grown at 5% CO_2_ and 37°C (Van Hoorde et al., 2000).

### Tissues Collection

After 15 days of walking treatment or *A. muciniphila* supplementation treatment, the C57BL/6J mice were sacrificed to assess the inflammation of the intestine. The length of colon was measured (Xiao et al., 2020), and small intestine tissue was removed for RNA isolation, protein extraction, and histological staining.

### Quantification of the Expression of IL-1β, IL-6, TNF-α, and Reactive Oxygen Species by ELISA

Small intestine tissues in each experimental group were ground with 200 μl of saline, followed by centrifugation for 10 min at 6,000 rpm and 4°C. Protein levels were measured from the clear supernatant using ELISA kit (Mlbio, Shanghai, China) according to the manufacturer’s instructions. Optical density was read at 450 nm (Rayto, Shenzhen, China).

### RNA Isolation and Quantitative Reverse Transcription Real-Time PCR

Total RNA was extracted from intestine tissues using TRIzol reagent (Invitrogen, Carlsbad, CA, United States) according to the manufacturer’s protocol. Complementary DNA was synthesized from total RNA using poly(A)-tailed total RNA and reverse transcription primer with ImPro-II Reverse Transcriptase (Promega, Madison, WI, United States), according to the manufacturer’s protocol. The qRT-PCR was performed according to the instructions of Fast Start Universal SYBR Green Master (Rox) (Roche Diagnostics GmbH, Mannheim, Germany). Experiments were conducted in duplicate in three independent assays. Relative transcriptional folds were calculated as 2^–ΔΔCt^. GAPDH was used as an internal control for normalization. All primers are listed in Supplementary Table 1.

### Histology

Following euthanasia, small intestine tissues of C57BL/6J mice were fixed in 4% buffered formalin overnight at room temperature and then embedded in paraffin. Tissues were sectioned at 5-μm thickness and co-stained with hematoxylin and eosin (H&E) using HE Staining Kit (Solarbio, Beijing, China) (Cardiff et al., 2014). For periodic acid–Schiff (PAS) staining, the small intestines of mice were fixed in Carnoy’s solution (absolute ethanol:chloroform:glacial acetic acid = 6:3:1) for 3 h. Dewaxed sections were hydrated and incubated in 1% periodic acid for 10 min followed by incubation in Schiff’s reagent for 10 min. Sections were counterstained with Mayer’s hematoxylin for 30 s and then washed and dehydrated before mounting with Pertex. Immunohistochemical (IHC) staining of tumor samples from BALB/c athymic nude mice were performed as previously reported (Xiao et al., 2020); and the primary antibody of rabbit anti-Ki-67 (Proteintech Group, Chicago, IL, United States) was used. Categorization of immunostaining intensity was performed by three independent pathologists. Sections were examined under light microscopy.

### *In vivo* Tumor Xenograft Assay

HCT-8 (or A549) cells were harvested and suspended at 2 × 10^7^ per ml with sterile normal saline. Groups of 4-week-old-male nude mice were subcutaneously injected in the armpit with 200 μl of cell suspensions (Xiao et al., 2020). When the tumor volume reached approximately 100 mm^3^, the mice were divided into two groups randomly based on the sizes of the tumors (*n* = 8 per group) (Sack et al., 2011; Martín-Ruiz et al., 2020) and received 10 Gy of radiotherapy (2 Gy × 5 day) (Kogelnik and Withers, 1978; Xiao et al., 2020). For the irradiation, mice were positioned under a lead shield so that only the tumor area was exposed. The mice in TAI + W group maintained the walking treatment for 15 days following radiotherapy, while TAI group maintained fasting and water deprivation for the same time. Tumor growth was measured every 3 days. Tumor volume (V) was monitored by measuring the length (L) and width (W) with calipers and calculated with the formula *V* = (L × W^2^) × 0.5. After 15 days, tumor-bearing mice were sacrificed, and the tumors were excised and weighed.

### Bacterial Diversity Analysis

Stool samples were freshly collected from two independent experiments and stored at −80°C until use. DNA was extracted from the stool using the Power Fecal^®^ DNA Isolation Kit (MoBio, Carlsbad, CA, United States). The DNA was recovered with 30 ml of buffer in the kit. PCR products were mixed in equidensity ratios. Then, mixture PCR products were purified with Qiagen Gel Extraction Kit (Qiagen, Hilden, Germany). The 16S ribosomal RNA (rRNA) V4 gene was analyzed to evaluate the bacterial diversity using lonS5TMXL lon 530 Chip (Thermo Fisher, Waltham, MA, United States). Sequence analysis was performed by Uparse software (Uparse v7.0.1001)^2^. Sequences with ≥97% similarity were assigned to the same operational taxonomic units (OTUs). Representative sequence for each OTU was screened for further annotation. For each representative sequence, the Silva123 Database was used based on RDP classifier (Version 2.2)^3^ algorithm to annotate taxonomic information. Briefly, each cohort contains 16 mice, and four mice share one cage. For gut microbiota analysis, we collected two fecal pellets from each cage to avoid cage effects. The primers are listed in Supplementary Table 1.

### Untargeted Metabolomics–Metabolite Extraction

Feces were individually grounded with liquid nitrogen, and the homogenate was suspended with prechilled 80% methanol and 0.1% formic acid (FA) by well vortexing. The samples were incubated on ice for 5 min and then were centrifuged at 15,000 rpm, 4°C for 5 min. Some of supernatant was diluted to final concentration containing 60% methanol by liquid chromatography–MS (LC-MS)-grade water. The samples were subsequently transferred to a fresh Eppendorf tube with 0.22-μm filter and then were centrifuged at 15,000 *g*, 4°C, for 10 min. Finally, the filtrate was injected into the LC-MS/MS system analysis (Xiao et al., 2020).

### Untargeted Metabolomics—Ultra-High-Performance Liquid Chromatography–MS/MS Analysis

LC-MS/MS analyses were performed using a Vanquish UHPLC system (Thermo Fisher, Waltham, MA, United States) coupled with an Orbitrap Q Exactive HF-X mass spectrometer (Thermo Fisher, Waltham, MA, United States). Samples were injected into a Hyperil Gold column (100 × 2.1 mm, 1.9 μm) using a 16-min linear gradient at a flow rate of 0.2 ml/min. The eluents for the positive polarity mode were eluent A (0.1% FA in water) and eluent B (methanol). The eluents for the negative polarity mode were eluent A (5 mM of ammonium acetate, pH 9.0) and eluent B (methanol). The solvent gradient was set as follows: 2% B, 1.5 min; 2%–100% B, 12.0 min; 100% B, 14.0 min; 100%–2% B, 14.1 min; and 2% B, 16 min. Q Exactive HF-X mass spectrometer was operated in positive/negative polarity mode with spray voltage of 3.2 kV, capillary temperature of 320°C, sheath gas flow rate of 35 arb, and aux gas flow rate of 10 arb.

### Untargeted Metabolomics—Data Analysis

The raw data files generated by UHPLC-MS/MS were processed using the Compound Discoverer 3.0 (CD 3.0, Thermo Fisher, Waltham, MA, United States) to perform peak alignment, peak picking, and quantitation for each metabolite. The main parameters were set as follows: retention time tolerance, 0.2 min; actual mass tolerance, 5 ppm; signal intensity tolerance, 30%; signal/noise ratio, 3; and minimum intensity, 100,000. After that, peak intensities were normalized to the total spectral intensity. The normalized data were used to predict the molecular formula based on additive ions, molecular ion peaks, and fragment ions. And then peaks were matched with the mzCloud^4^ and ChemSpider^5^ database to obtain the accurate qualitative and relative quantitative results.

### Statistical Analysis

Each experiment was repeated at least three times. Data were assessed with normal distribution using the Kolmogorov–Smirnov test. The data are presented as the means ± SD with respect to the number of samples (n) in each group. Significance was assessed by comparing the mean values using Student’s *t*-test and Wilcoxon rank sum test for independent groups, as follows: ^∗^*p* < 0.05; ^∗∗^*p* < 0.01; and ^∗∗∗^*p* < 0.005. Results with *p* < 0.05 were considered statistically significant.

## Results

### Walking Alleviates Radiation-Induced Gastrointestinal Tract Injuries in Male Mice

**FIGURE 1:**
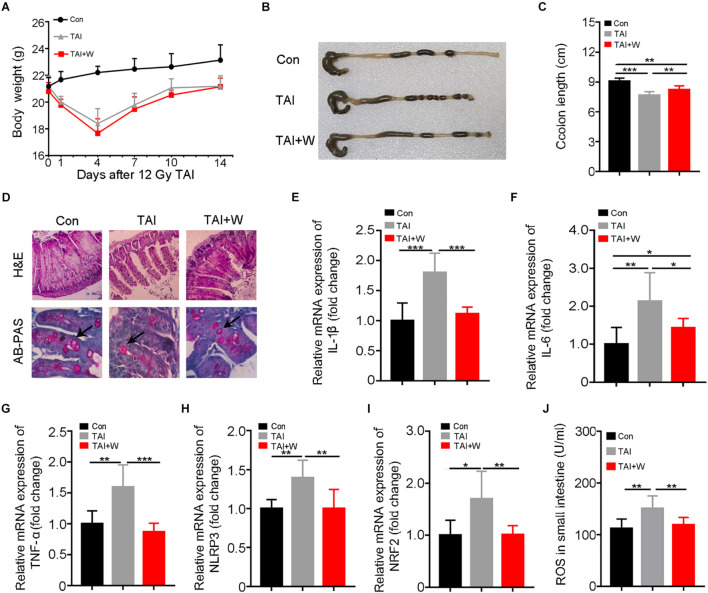
Walking as a non-pharmacological regimen alleviates radiation-induced GI tract injuries in male mice. Male mice were exposed to 12 Gy of TAI, and TAI + W group maintained walking for 15 days, and TAI group had no treatment. Then the colon and small intestine tissues were obtained at day 16, *n* = 10 per group. **(A)** The body weight of male mice in the three groups. **(B)** Photographs of dissected colon from male mice in the three groups. **(C)** Statistical results of colon length between two groups. **(D)** The morphology of the small intestine from male mice in the three groups is shown by H&E and PAS staining. The black arrows point to the goblet cells. **(E–I)** The expression levels of *IL-1*β, *IL-6*, *TNF-*α, *NLRP3*, and *NRF2* were examined in small intestine tissues from male mice by qRT-PCR. **(J)** The levels of ROS in the small intestine of male mice were measured by ELISA. Significant differences are indicated: *p < 0.05, **p < 0.01, and ***p < 0.005 by Student’s *t*-test between two cohorts. GI, gastrointestinal; TAI, total abdominal irradiation; W, walking; PAS, periodic acid–Schiff; ROS, reactive oxygen species.

All experimental mice were exposed to abdomen local irradiation. Then, one cohort of mice was enforced to walk (35 min of 1 m/min, Supplementary Figure 1) for 2 weeks. As shown in Figure 1A, walking did not change the body weight loss of irradiated mice. To address the effects of walking on radiation-induced GI toxicity, we assessed the colon length and histological structure of the small intestines. Intriguingly, mice with walking treatment had longer colon (Figures 1B,C), denser small intestinal villi, and more goblet cells (Figure 1D) than did the mice with irradiation only. In addition, abdominal radiation stimuli elicited the syndromes for enteritis, as judged by the upregulation of inflammatory factors expression in the small intestine; however, short-term walking erased the elevation (Supplementary Figure 2; Figures 1E–I). Meanwhile, walking reduced the level of intestinal oxidative stress following ionizing radiation (Figures 1I,J). Together, our findings demonstrate that walking as a form of low-intensity exercise protects against radiation-induced GI tract toxicity in male mice.

### Walking Reshapes Gut Microbiota Configuration Following Radiation Challenge

**FIGURE 2:**
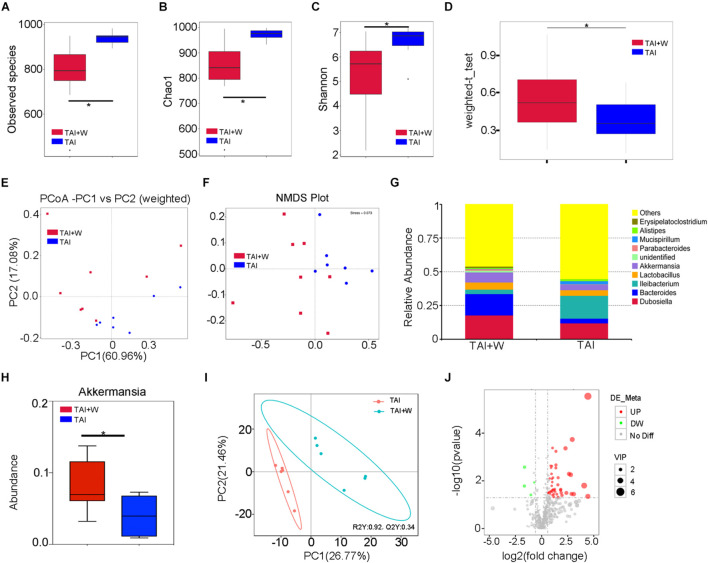
Walking reshapes gut microbiota configuration following radiation challenge. The gut bacterial composition structures in male mice of TAI (radiation alone) and TAI + W (walking after radiation) groups were measured by 16S rRNA high-throughput sequencing at 16 days following TAI exposure, *n* = 7 (TAI group) or *n* = 8 (TAI + W groups). The gut metabolite composition was detected by untargeted metabolomics at 16 days following TAI exposure, *n* = 6 (TAI group) or *n* = 6 (TAI + W groups). **(A–C)** Alpha diversity was measured: **(A)** the observed species number, **(B)** Chao1 diversity index, and **(C)** Shannon diversity index. **(D)** The beta diversity of intestinal bacteria was compared by weighted *t*-test analysis. **(E,F)** PCoA (weighted) and NMDS were performed to assess the alteration of gut bacteria taxonomic profile from male mice in two groups. **(G)** The relative abundances of the top 10 varied strain bacteria at the genus level in male mice of two groups. **(H)** The abundance of *Akkermansia* in male mice of two groups. **(I)** The PLSDA score of positive metabolites in feces in the two groups. **(J)** The volcano diagram of positive metabolites in feces. **(A–C)** Significant differences are indicated: Wilcoxon rank sum test. **(D,H)** Significant differences are indicated: *p < 0.05 by Student’s *t*-test between two cohorts. TAI, total abdominal irradiation; W, walking; PCoA, principal coordinates analysis; NMDS, non-metric multidimensional scaling; PLSDA, partial least-squares discriminant analysis.

Given the close relationship between the gut microbiota and the radiation-induced GI tract toxicity, we performed 16S rRNA gene amplicon surveys to analyze the bacterial composition in fecal pellets from abdomen local irradiated mice with or without short-term walking treatment. As shown in Figures 2A–C, 2 weeks of walking treatment decreased the alpha diversity of gut flora in the irradiated mice. Reversely, the beta diversity of microorganism in droppings was increased from the mice with walking treatment (Figure 2D; Supplementary Figures 3A–C). Weighted principal coordinates analysis (PCoA) and non-metric multidimensional scaling (NMDS) further exhibited an obvious separation of enteric bacteria between the two cohorts, indicating that walking indeed remolds the radiation-shifted intestinal bacterial profile (Figures 2E,F). In detail, the mice with short-term walking treatment showed a predominance of *Dubosiella*, *Bacteroides*, *Akkermansia*, and *Lactobacillus* at the genus level (Figures 2G,H; Supplementary Figures 3D–F). Next, the analysis of metabolome of gut microbiome showed that walking propelled a variation in metabolite profile (Figures 2I,J; Supplementary Figures 3G,H), indicating that walking not only alters the gut microbiota structure but also impacts the function of gut flora. Together, our observations demonstrate that short-term walking restructures the gut microbiome after radiation exposure.

### Walking Mitigates Intestinal Radiation Toxicity Depending on Gut Microbiota

To find out whether walking mitigating radiation-induced GI tract toxicity depends on gut microbiota, an antibiotic cocktail (ABX) was added in the drinking water to remove the gut microbes of the irradiated mice. Intriguingly, ABX treatment hindered the radioprotection of walking toward GI injuries, as judged by shortening colon, loss of intestinal villi, reduced goblet cells, and elevated inflammatory status (Supplementary Figure 4), implying that gut microbiota might contribute to the radioprotection of short-term walking.

**FIGURE 3:**
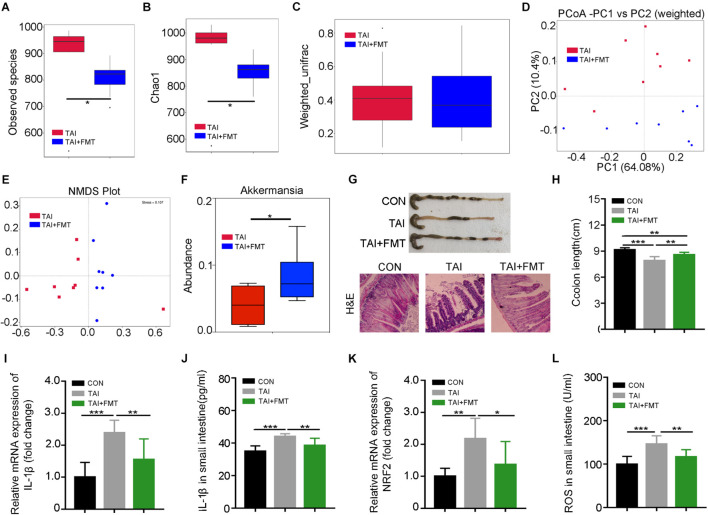
Walking mitigates intestinal radiation toxicity depending on gut microbiota. Male mice were exposed to 12 Gy of TAI, and for TAI + FMT group, irradiated mice were administrated with fecal microbiota via oral route from male donors that maintained walking following radiation exposure. The gut bacterial composition structures in male mice of TAI and TAI + FMT groups were measured by 16S rRNA high-throughput sequencing at 16 days after TAI exposure, *n* = 8 per group. The colon and small intestine tissues were obtained at day 16, *n* = 10 per group. **(A,B)** Alpha diversity was measured: **(A)** the observed species number and **(B)** Chao1 diversity index. **(C)** The beta diversity of intestinal bacteria. **(D,E)** PCoA (weighted) and NMDS were performed to assess the alteration of gut bacteria taxonomic profile from male mice in two groups. **(F)** The abundance of *Akkermansia* in male mice of two groups. **(G)** Photographs of dissected colon and morphology of the small intestine shown by H&E from male mice in the two groups. **(H)** Statistical results of colon length between two groups. **(I,K)** The expression levels of *IL-1*β and *NRF2* were examined in small intestine tissues from male mice by qRT-PCR. **(J,L)** The levels of *IL-1*β and ROS in the small intestine of male mice were measured by ELISA. **(A–C)** Significant differences are indicated: Wilcoxon rank sum test. **(D–L)** Significant differences are indicated: *p < 0.05, **p < 0.01, and ***p < 0.005 by Student’s *t*-test between two cohorts. TAI, total abdominal irradiation; FMT, fecal microbiota transplantation; PCoA, principal coordinates analysis; NMDS, non-metric multidimensional scaling; ROS, reactive oxygen species.

Next, FMT was performed to further validate the roles of gut microbiota in the system. The donor mice walked for 2 weeks after abdomen local irradiation (Supplementary Figure 5). Same with the donors, the alpha diversity of gut bacteria in irradiated recipients declined as compared with that of the mice with irradiation only (Figures 3A,B). Although weighted_unifrac analysis showed no change of the enteric microbiota statistically (Figure 3C), PCoA and NMDS plot was conducted to visualize differences in bacterial taxa composition between the two groups (Figures 3C–E; Supplementary Figure 6D). In parallel, FMT caused an enrichment on *Dubosiella*, *Bacteroides*, *Akkermansia*, and *Lactobacillus* at the genus level in recipients compared with saline-treated controls (Figure 3F; Supplementary Figures 6E–G), indicating the gut microbiota community of recipients is shifted and similar to that of the donors after FMT. Consistent with the aforementioned results, recipients shared the same dynamic changes of body weight to irradiated controls (Supplementary Figure 6H) and had longer colon (Figure 3G), denser intestinal villi, more goblet cells (Figure 3H), lower proinflammatory cytokine levels, and fewer reactive oxygen species (ROS) production than in saline treatment (Figures 3I–L; Supplementary Figures 6I–K). Together, our observations corroborate that walking fights against radiation-induced GI tract injuries at least partly depending on modulating gut microbiota.

### *Akkermansia muciniphila* Mitigates Radiation-Elevated Inflammatory Status in Digestive Tract

**FIGURE 4:**
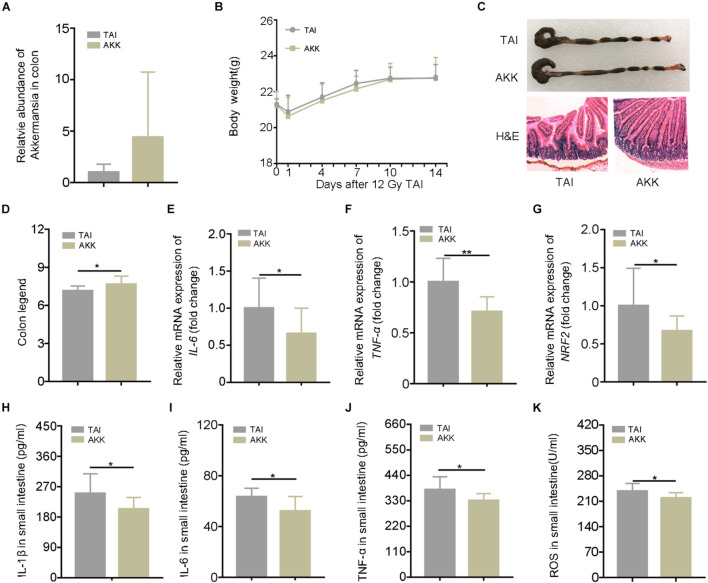
*Akkermansia muciniphila* (AKK) mitigates radiation-elevated inflammatory status in digestive tract. Male mice were exposed to 12 Gy of TAI; and for AKK group, the male mice were orally administrated with *A. muciniphila* (1.5 * 10^8^ CFU) for 15 days following radiation challenge, *n* = 10. **(A)** The relative abundance of *A. muciniphila* in the colon was detected by q-PCR. **(B)** The body weight of male mice in the two groups. **(C)** Photographs of dissected colon and morphology of the small intestine shown by H&E from male mice in the two groups. **(D)** Statistical results of colon length between two groups. **(E–G)** The expression levels of *IL-6*, *TNF-*α, and *NRF2* were examined in small intestine tissues from male mice by qRT-PCR. **(H–K)** The levels of *IL-1*β, *IL-6*, *TNF-*α, and ROS in the small intestine of male mice were measured by ELISA. Significant differences are indicated: *p < 0.05, and **p < 0.01 by Student’s *t*-test between two cohorts. TAI, total abdominal irradiation; ROS, reactive oxygen species.

The 16S rRNA sequencing analysis showed a predominance of *A. muciniphila* at the genus level following walking and FMT experiments. In addition, *A. muciniphila* has been reported to be negative correlation with numerous diseases including IBDs, and the frequency of the bacteria was increased in dextran sodium sulfate (DSS) mice with running treatment. Thus, we speculated that as a potential probiotic, *A. muciniphila* might be the key element for the radioprotection of walking. The abdominal irradiated mice were treated with *A. muciniphila* (1.5 × 10^8^ CFU) via oral route for 15 days. As shown in Figure 4A, the relative abundance of *A. muciniphila* in feces increased after the treatment, validating that *A. muciniphila* was colonized in the intestinal tract of mice successfully. Although oral gavage of *A. muciniphila* did not change the body weight of irradiated mice (Figure 4B), the length of colon and the integrity of intestinal villi were improved (Figures 4C,D; Supplementary Figure 7A). In addition, *A. muciniphila* treatment reduced the levels of inflammatory markers in the small intestine (Figures 4E–K; Supplementary Figure 7B), suggesting that the enrichment of *A. muciniphila* in lower digestive tract ameliorates radiation-induced enteritis. Together, our observations demonstrate that *A. muciniphila* might bolster the radioprotection of walking treatment.

### Walking Fights Against Radiation-Induced Gastrointestinal Tract Toxicity in Female Mice

**FIGURE 5:**
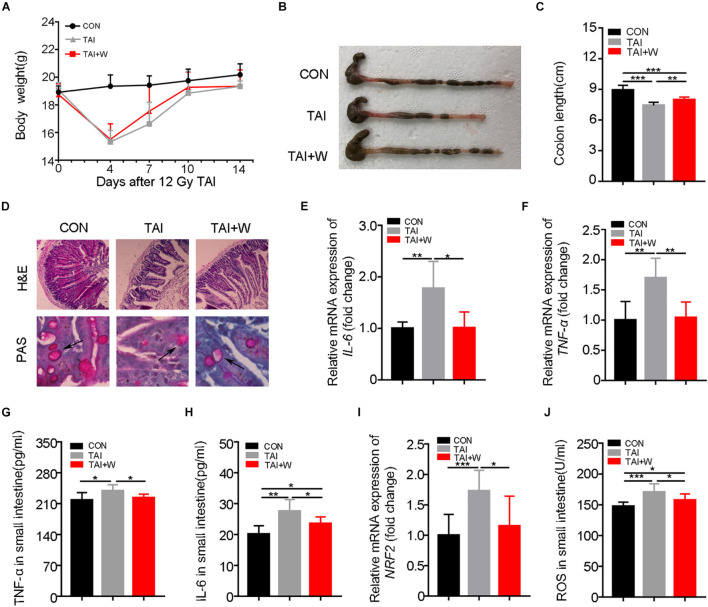
Walking protects against radiation-induced GI tract toxicity in female mice. Except for female mice in Con group, female mice of the other two groups were exposed to 12 Gy of TAI, TAI + W group maintained walking for 15 days, and TAI group had no treatment. **(A)** The body weight of male mice in the three groups. **(B)** Photographs of dissected colon from male mice in the two groups. **(C)** Statistical results of colon length between two groups. **(D)** The morphology of the small intestine from male mice in the two groups was shown by H&E and PAS staining. The black arrows point to the goblet cells. **(E–G)** The expression levels of *IL-6*, *TNF-*α, and *NRF2* were examined in small intestine tissues from male mice by qRT-PCR. **(H–J)** The levels of IL-6, TNF-α, and ROS in the small intestine of male mice were measured by ELISA. Significant differences are indicated: *p < 0.05, **p < 0.01, and ***p < 0.005 by Student’s *t*-test between two cohorts. GI, gastrointestinal; Con, control; TAI, total abdominal irradiation; W, walking; PAS, periodic acid–Schiff.

Given that sexual dimorphism affected the treatment efficacy in radiation syndrome (Cui et al., 2019), we elucidated whether walking as a non-pharmacological remedy can be used to protect females against GI tract toxicity following irradiation. Same to the male counterparts, abdomen local irradiated female mice with short-term walking treatment did not show further weight loss (Figure 5A). As expected, the female mice with walking treatment had longer colons, denser small intestinal villi, and more goblet cells (Figures 5B–D). ELISA and qRT-PCR assays revealed that walking reduced the expression of inflammatory factors (Figures 5E–H; Supplementary Figures 8A,B) and the level of oxidative stress (Figures 5I,J). Together, our findings indicate that walking is an efficacious strategy to mitigate radiation-induced GI tract toxicity in both male and female mice.

### Walking Does Not Alter the Proliferation of Cancer Cells Following Radiation Exposure

**FIGURE 6:**
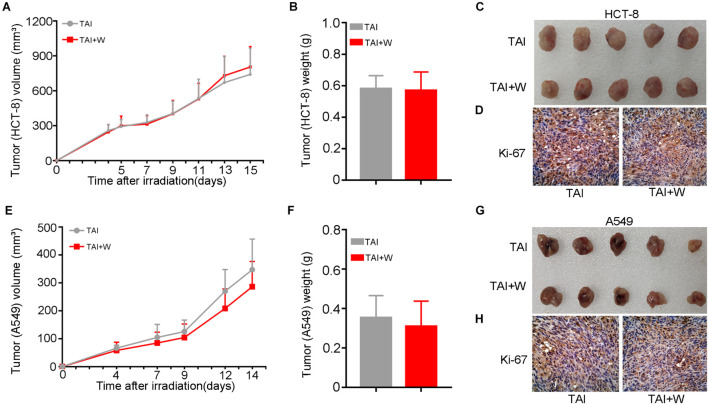
Walking did not alter the proliferation of cancer cells following radiation exposure. We subcutaneously injected HCT-8 or A549 tumor cells into the armpit of nude mice; then the mice received 10 Gy of radiotherapy (2 Gy * 5 days) when the tumors reached 100 mm^3^. The growth of tumors was observed in mice with walking (TAI + W) or not (TAI). The tumor volume was recorded during the tumorigenicity process every 2 or 3 days, *n* = 8 per group. **(A–C)** The growth curves of tumors, tumor weights, and representative image of excised tumors of HCT-8 group. **(D)** The expression levels of Ki-67 in HCT-8 tumor tissues were examined by IHC staining. **(E–G)** The growth curves of tumors, tumor weights, and representative image of excised tumors of A549 group. **(H)** The expression levels of Ki-67 in A549 tumor tissues were examined by IHC staining. GI, gastrointestinal; Con, control; TAI, total abdominal irradiation; W, walking; PAS, periodic acid–Schiff; IHC, immunohistochemical.

To find out the safety of walking for cancer patients with radiotherapy, we injected HCT-8 (or A549) cells into nude mice subcutaneously and recorded the tumor growth after local radiation stimuli with or without walking treatment. Intriguingly, walking did not change the proliferation of HCT-8 (or A549) cells and the terminal weight of the xenografts (Figures 6A–C,E–G). Then we tested the expression of Ki-67, a cell proliferative marker, in the tumor tissues from the two groups. IHC assay revealed that walking did not alter the expression of Ki-67, further indicating that walking does not interfere with the tumoricidal effects of radiation therapy (Figures 6D,H).

## Discussion

Radiotherapy as a milestone for oncotherapy is applied to more than 50% of the global cancer patients, but the radiation toxicity of the whole body, especially for adjacent organs, is urgently worrisome (Citrin, 2017; De Ruysscher et al., 2019). For example, abdomen local irradiation, as a common means for treating abdominopelvic tumor, always leads harm to the hematopoietic system, GI tract, and even reproductive system, which is near the exposed area (Rappleye et al., 1975; Zhang et al., 2016; Oktem et al., 2018). As an organ within the treatment field for all intra-abdominal, retroperitoneal, and pelvic tumors, the intestine gets interfered unavoidably during or after radiotherapy, manifesting in acute (or chronic) inflammation, apoptosis, and fibrosis (Wei et al., 2016). In the United Kingdom, about 90% of patients receiving pelvic radiation reported alterations in their bowel function, which leads to negative effects on daily activity in up to 50% (Andreyev, 2007; Takemura et al., 2018). Despite great advancement in delivery technology of radiotherapy [e.g., FLASH-RT and accelerated partial breast irradiation (APBI)], these adverse side effects remain an overwhelming medical challenge (Vozenin et al., 2019; Whelan et al., 2019). In addition, mounting evidence proves that proinflammatory cytokines, such as interleukin-1β, interleukin-18, and inflammatory CC chemokines, are associated with carcinogenesis (Vetrano et al., 2010; Chen and Núñez, 2011). The chronic proinflammatory state of intestine hijacking immune system precipitates tumor outgrowth (Biragyn and Ferrucci, 2018; Olafsson et al., 2020). All the reports highlight the ill effects of intestinal inflammation in tumorigenesis and oncotherapy. Importantly, there are no safe and effective therapeutic approaches to overcome intestinal radiation injury currently, and some studies show that the secondary reactions of the corresponding drugs reduce patient tolerability and even interrupt the treatment (Rios et al., 2014; Lawrie et al., 2018). In this study, we observed that short-term walking, a low-intensity physical activity, drove milder GI tract toxicity, especially lower level of intestinal inflammation. The findings suggest that walking might be a rehabilitation maneuver for cancer patients with radiotherapy. Physical activities are identified as low, moderate, and vigorous intensities on the basis of those metabolic equivalents (Martínez et al., 1997). Different intensities of exercise elicit different responses (Marijon et al., 2011; Schnohr et al., 2015). Running as a form of vigorous exercise receives more attention; however, the intertwined adverse effects on knee and heart are still an issue (Whyte et al., 2008; Alentorn-Geli et al., 2017). Compared with running, walking is a more comfortable and feasible form of exercise and can be acceptable to weak patients undergoing radiotherapy. Many meta-analyses have revealed that walking reduces cardiovascular risk, governs body mass index, and regulates blood glucose and lipid (Hamer and Steptoe, 2008; Oja et al., 2018). In this study, walking did not cause weight loss in experimental mice, indicating that walking is a kind of safe exercise for cancer patients with radiotherapy. Importantly, due to the low-intensity and short exercise time per day, walking is suitable for almost all mobile cancer patients without economic burden. Sexual dimorphism impacts the curative effects and the prognosis of cancer patients (Cui et al., 2019). Thus, we collected the data from male and female mouse models in this study and reported that walking is applicable to cancer patients in both sexes to improve the prognosis following radiotherapy.

National Comprehensive Cancer Network (NCCN) Guidelines point out that radiotherapy is the optional remedy for pelvic and abdomen tumors including prostate cancer (early, middle, and late stages) and cervical cancer (invasive cancer of various stages) and thoracic tumors such as breast cancer (early, locally advanced and metastatic breast cancer). In this study, we identified that walking did not accelerate the proliferation of cancer cell in tumor xenograft models. Although the model cannot be used to evaluate the stage of cancers, we focused on the ameliorating effect of walking on radiotherapy-intertwined intestinal toxicity. Therefore, all the patients with radiotherapy suffering from GI tract syndrome could perform walking to mitigate the complications.

Millions of commensal microbes inhabit the GI tract of mammals and are involved in immune regulation and energy metabolism. Intestinal microorganism imbalance propels multiple diseases. More and more studies suggest that gut microbiota plays vital roles in intestinal radiation injury (Touchefeu et al., 2014; Huang et al., 2019). Clinical trials identify that exercise indeed improves the prognosis of cancer patients; however, the underlying mechanism remains confusing (Wang et al., 2011; Bjerre et al., 2019; Lundt and Jentschke, 2019). In light of the close relationship between exercise and gut microbiota, the irradiated mice were treated with antibiotic cocktail or FMT in the present study. Gut flora deletion erased the radioprotection of walking, and recipients harboring gut microbes from donor with walking treatment exhibited milder GI tract injuries. All the results indicated that radioprotection of walking might be partly dependent on gut bacterial structure reorganization. Walking spurred an enrichment on some intestinal bacteria, such as *Akkermansia*, *Bacteroides*, *Dubosiella*, and *Lactobacillus*. *Bacteroides* shows an enrichment at the early stage after irradiation, implying that the bacteria might be a driver for radiation toxicity (Wang et al., 2019; Li et al., 2020b). Yet the effect of *Dubosiella* on enteritis is still controversial (Sheng et al., 2020). *Lactobacillus* as well-known probiotics have been proved to be associated with improvement in patients suffering from radiation syndrome in clinical trials (Linn et al., 2019). A recent study has reported that *A. muciniphila* improves intestinal radiation injury following whole body irradiation (Kim et al., 2021). Notably, the experimental mice in the present study were exposed to abdominal local irradiation, which is more similar to iatrogenic irradiation such as radiotherapy for pelvic and abdominal tumors. In addition, the published study focuses on the relationship between *A. muciniphila* supplement and the function of intestinal stem cells. Given that radiation-induced gastroenteritis and colitis are common adverse side effects in cancer patients with systemic therapy or radiotherapy (Jairam et al., 2019), we wondered whether *A. muciniphila* might be employed to protect against radiation intestinal inflammation in this study. We treated irradiated mice with *A. muciniphila* via oral route. *A. muciniphila* replenishment reduced inflammation levels and increased integrity of the small intestine after local radiation stimuli. The results bolster that *A. muciniphila* might be a potential probiotic for cancer patients with radiotherapy. However, the optimal use method of *A. muciniphila* in clinical application required further study.

**FIGURE 7:**
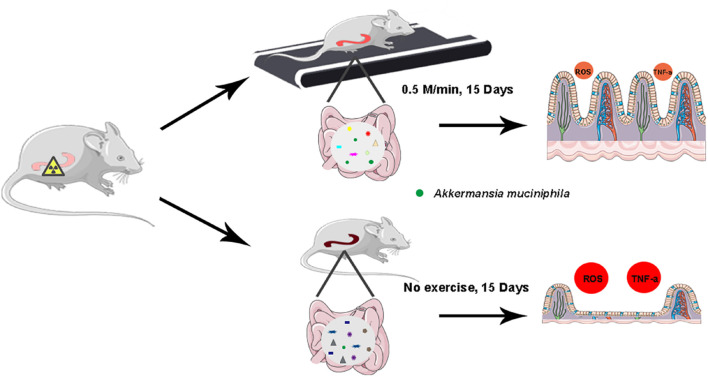
Illustration of walking for the alleviation of radiation-induced intestinal inflammation.

There are still some limitations requiring further study to pave the way for walking to be integrated into clinical application. Firstly, although the walking protocol ensures the quality and quantity of exercise, involuntary walking cannot be completely avoided. Secondly, the frequency and intensity of walking for patients with radiotherapy need to be further explored based on clinical trials. Finally, long-term low-intensity exercise has been proved to reduce the occurrence of CRC and stimulate tumor cell apoptosis (Kim et al., 2020), and our findings identify that 15-day walking treatment does not accelerate the growth of tumors. Therefore, long-term walking might be necessary to explore its effects on metastasis of cancers. In conclusion, walking as a low-intensity physical activity alleviates intestinal radiation toxicity in both male and female mice. Mechanistically, walking remolded the gut microbiota configuration and reprogrammed the intestinal microbial metabolome of abdomen local irradiated mice. *A. muciniphila*, a potential probiotic, might be employed to fight against radiation-induced GI tract injuries (Figure 7). Together, our observations provide new insights into the function of walking and underpin that walking is a safe and effective form to improve the prognosis of cancer patients with radiotherapy suffering from GI tract syndrome without financial burden in a preclinical setting.

## Data Availability Statement

The datasets presented in this study can be found in online repositories. The names of the repository/repositories and accession number(s) can be found below: MetaboLights, and the accession number is MTBLS2826.

## Ethics Statement

The animal study was reviewed and approved by animal experiments were performed according to the institutional guidelines approved by the Animal Care and Ethics Committee of IRM-PUMC.

## Author Contributions

BW, Y-XJ, and MC designed the experiments, analyzed the data, and wrote the manuscript. BW and Y-XJ performed the experiments and wrote the manuscript. MC provided writing assistance. S-QZ, H-WX, J-LD, Y-XJ, and YL proofread the article. MC, X-DY, and S-JF oversaw the entire project. All authors contributed to the article and approved the submitted version.

## Conflict of Interest

The authors declare that the research was conducted in the absence of any commercial or financial relationships that could be construed as a potential conflict of interest.

## Publisher’s Note

All claims expressed in this article are solely those of the authors and do not necessarily represent those of their affiliated organizations, or those of the publisher, the editors and the reviewers. Any product that may be evaluated in this article, or claim that may be made by its manufacturer, is not guaranteed or endorsed by the publisher.

## Footnotes

1
https://gco.iarc.fr/tomorrow/home

2
http://drive5.com/uparse/

3
http://sourceforge.net/projects/rdpclassifier/

4
https://www.mzcloud.org/

5
http://www.chemspider.com/
