# Supplementary material for: Low-Intensity Exercise Modulates Gut Microbiota to Fight Against Radiation-Induced Gut Toxicity in Mouse Models
Source: Front Cell Dev Biol. 2021 Oct 21;9:706755. doi: 10.3389/fcell.2021.706755 (PMC8566984; doi:10.3389/fcell.2021.706755)
Supplement: Supplementary file 1 [file Data_Sheet_1.docx]

***Supplementary materials***

**1 Supplementary Tables**

**Table. S1**

| **Gene** | **Primer** | **Sequence (5′-3′)** |
| --- | --- | --- |
| **Primers for PCR** | |  |
| *GAPDH* | forward | TGTTTCCTCGTCCCGTAGA |
|  | reverse | CAATCTCCACTTTGCCACTG |
| *IL-1β* | forward | TTGAAGAAGAGCCCATCCTC |
|  | reverse | CAGCTCATATGGGTCCGAC |
| *IL-6* | forward | TGTGCAATGGCAATTCTGAT |
|  | reverse | GGTACTCCAGAAGACCAGAGGA |
| *TNFɑ* | forward | TTCTCATTCCTGCTTGTGGCA |
|  | reverse | ACTTGGTGGTTTGCTACGACG |
| *NLRP3* | forward | ATCAACAGGCGAGACCTCTG |
|  | reverse | GTCCTCCTGGCATACCATAGA |
| *NRF2* | forward | ACAGTGCTCCTATGCGTGAAT |
|  | reverse | AAGCGGCTTGAATGTTTGTC |
| **Primers for sequencing** | | |
| 515F |  | GTGCCAGCMGCCGCGGTAA |
| 806R |  | GGACTACHVGGGTWTCTAAT |
| **16S rRNA Primers for PCR** | | |
| *Bacterial universal* | forward | ACTCCTACGGGAGGCAGCAG |
|  | reverse | ATTACCGCGGCTGCTGG |
| *A. muciniphila* | forward | CAGCACGTGAAGGTGGGGAC |
|  | reverse | CCTTGCGGTTGGCTTCAGAT |

**2 Supplementary Figures**


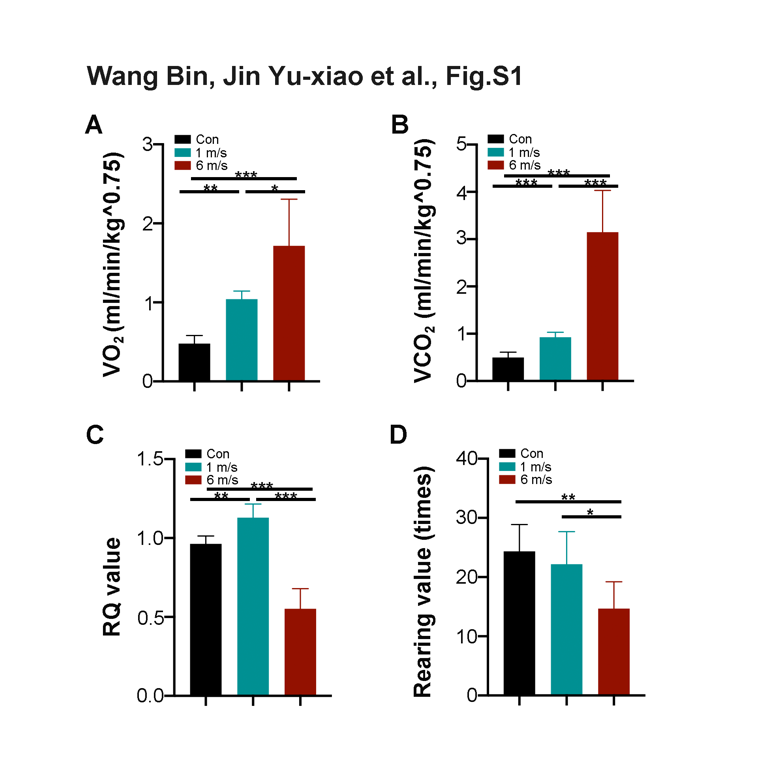


**Fig S1.** Slow walking is more suitable for frail mice. (A, B) CO_2_ exhalation (A) and O_2_ consumption (B) of mice every 15 min after walking with indicated speed (within half an hour). (C) The RQ value of mice every 15 min after walking with indicated speed (within half an hour). (D) The number of times the mouse rearing every 3 min (when the mouse exceeds the specified height, it is considered to be upright).


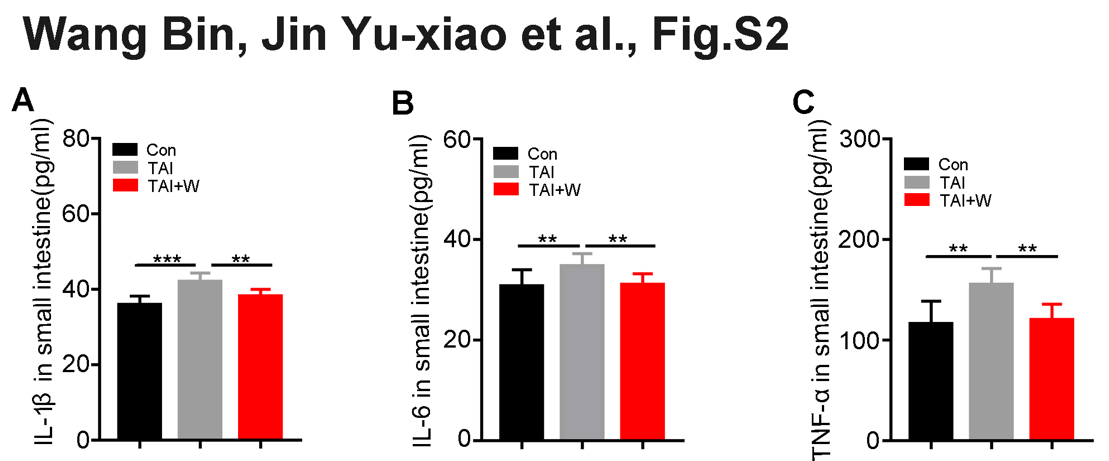


**Fig S2.** Walking ameliorates GI tract function and reduces inflammation response after irradiation. (A-C) The levels of *IL-1β*, *IL-6* and *TNF-α* in small intestine of male mice were measured by ELISA. Significant differences are indicated: ***P* < 0.01, ****P* < 0.005 by Student’s *t*-test between each two cohorts.


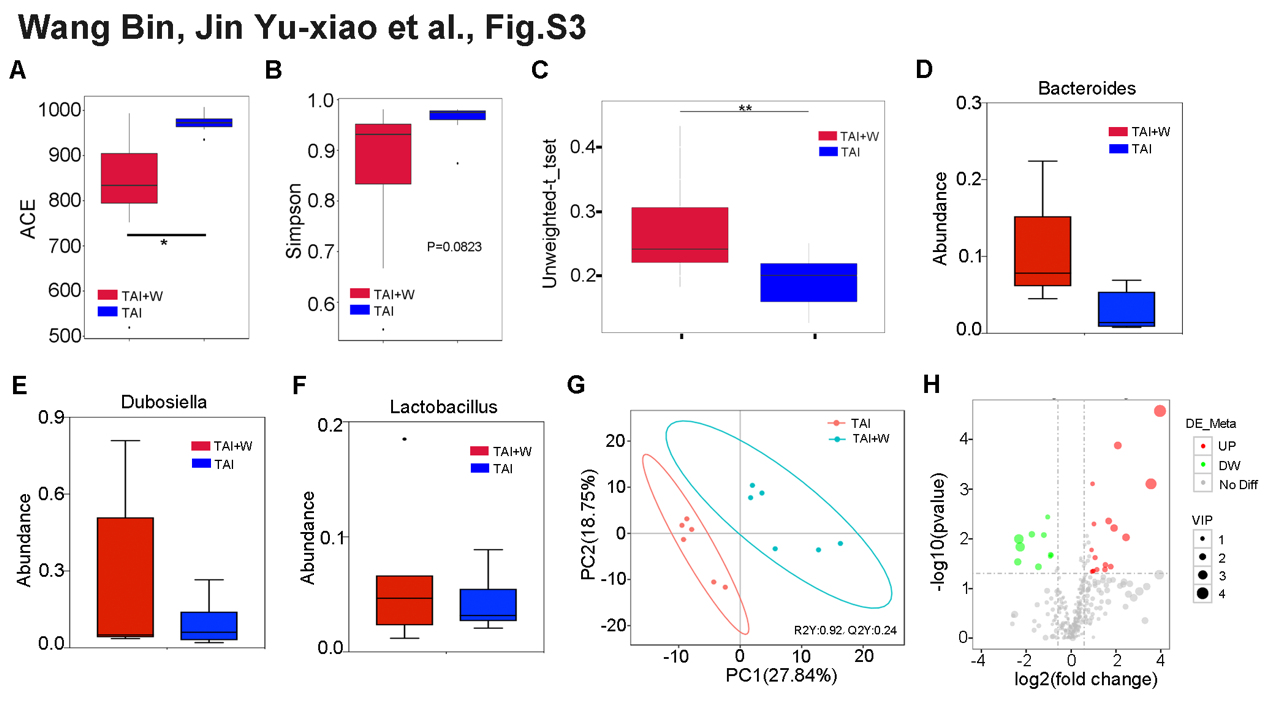


**Fig S3.** Walking reshapes intestinal bacterial structure and alters metabolite composition at day 16 after TAI. The gut bacterial composition structures in male mice of TAI and TAI+W groups were measured by 16S rRNA high-throughput sequencing at 16 d following TAI exposure, n = 7 (TAI group) or 8 (TAI+W groups). The gut metabolite composition was detected by untargeted metabolomics at 16 d following TAI exposure, n = 6 (TAI group) or 6 (TAI+W groups). (A and B) alpha diversity was measured: (A) ACE diversity index and (B) Simpson diversity index. (C) The beta diversity of intestinal bacteria was compared by unweighted t-test analysis (D-F) The abundance of *Bacteroides*, *Dubosiella* and *Lactobacillus* at the genus level in male mice gut of two groups. (G) The PLSDA-score of negative metabolites in feces in the two groups. (H) The volcano diagram of negative metabolites in mice of the two groups. Significant differences are indicated: **P* < 0.05 by Student’s *t*-test between two cohorts.


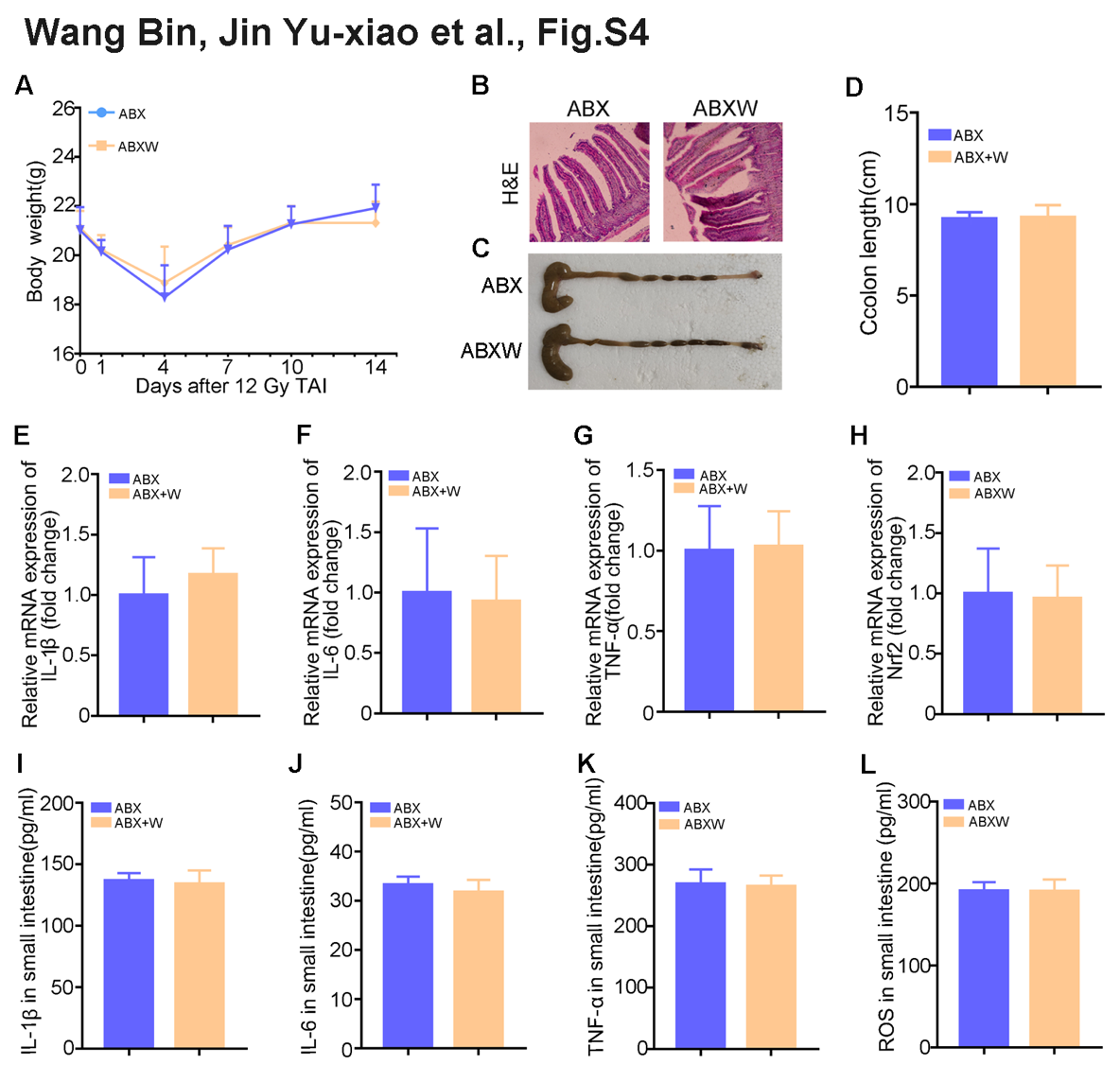


**Fig S4.** The improvement effect of walking on radiation-induced gastrointestinal injury is removed with the elimination of gut bacteria. For ABX group, mice in experimental group were housed with drinking water supplemented with ABX, an antibiotic cocktail that can subvert existing gut microbes, while ABXW group kept walking during ABX treatment. The colon and small intestine tissues were obtained at day 16, n = 10 per group. (A) The body weight of male mice in ABX and ABXW groups. (B) The morphology of the small intestines from male mice in the two groups was shown by H&E. (C) Photographs of dissected colon from male mice in the two groups. (D) Statistical results of colon length between two groups. (E-H) The expression level of *IL-1*β*, IL-6, TNF-*α and *NRF2* were examined in small intestine tissues from male mice by qRT-PCR. (I-L) The levels of *IL-1β*, *IL-6*, *TNF-α* and ROS in small intestine of male mice were measured by ELISA.


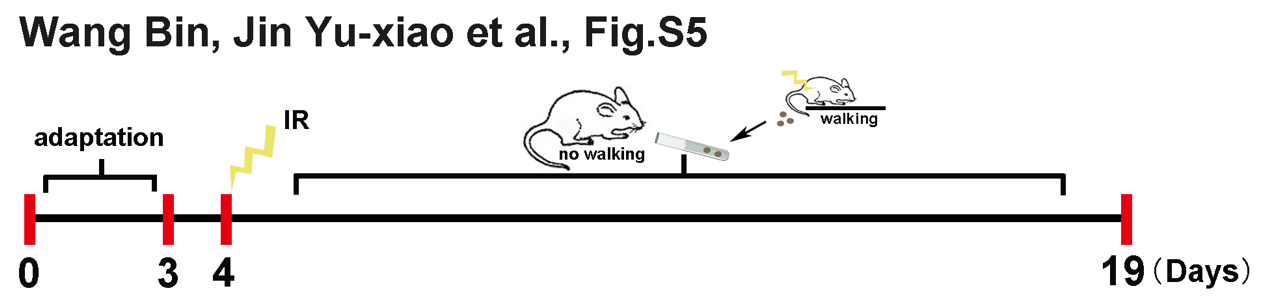


**Fig S5.** Schematic diagram of fecal microbiota transplantation treatment.


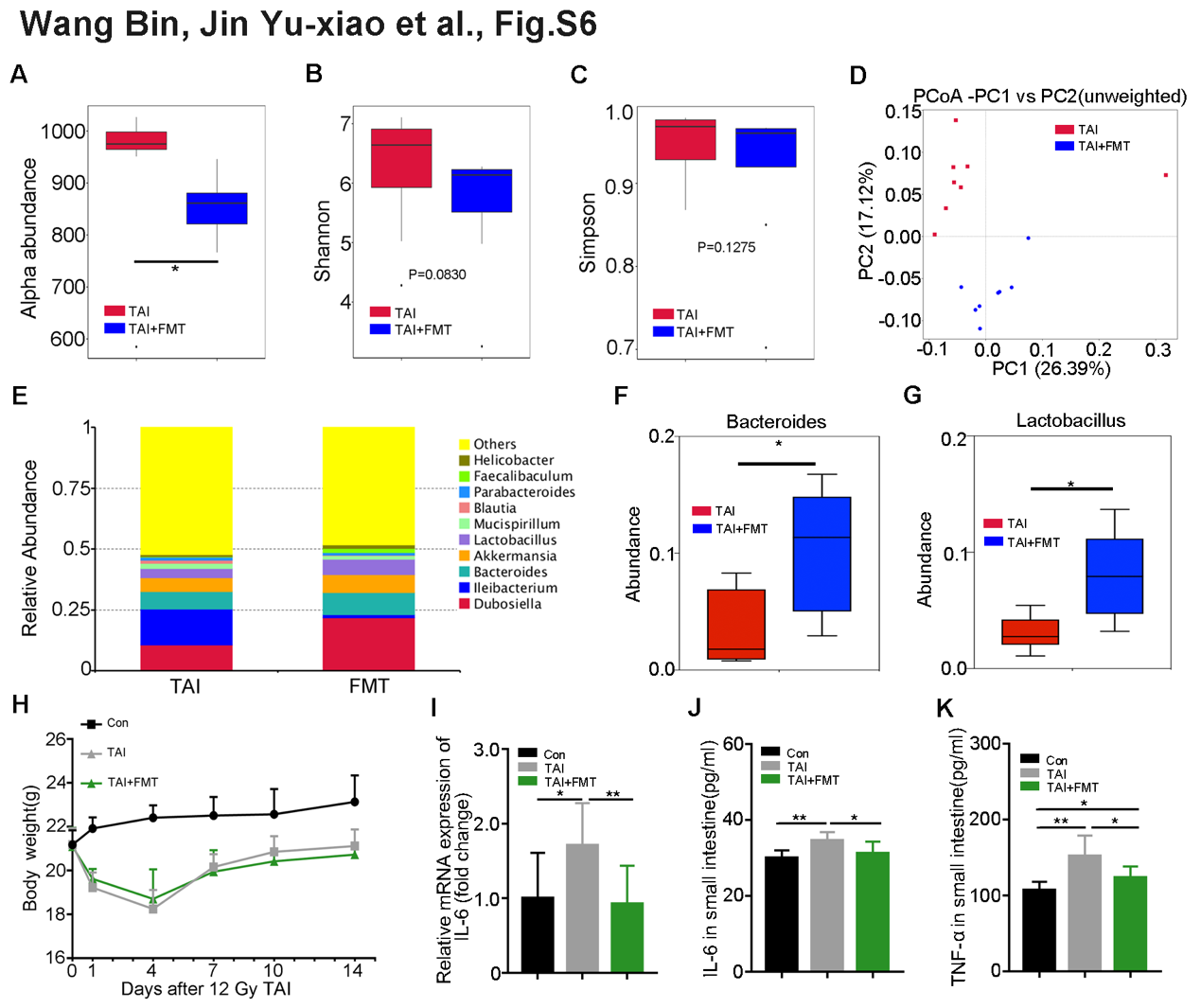


**Fig S6.** Walking plays a role in radiation intestinal injury by modifying gut microbiota composition. Male mice were exposed to 12 Gy total abdominal irradiation, and for FMT group, irradiation mice were treated with fecal microbiota *via* oral route from male donors which maintained walking following radiation exposure. The gut bacterial composition structures in male mice of TAI and TAI+FMT groups were measured by 16S rRNA high-throughput sequencing at 16 d after TAI exposure, n = 8 per group. The colon and small intestine tissues were obtained at day 16, n = 10 per group. (A-C) alpha diversity was measured: (A) Alpha abundance, (B) Shannon diversity index and (C) Simpson diversity index. (D) Unweighted PCoA was performed to assess the alteration of gut bacteria taxonomic profile from male mice in two groups. (E) The relative abundances of top10 varied strain bacteria at the genus level in male mice of two groups. (F and G) The abundance of *Bacteroides* and *Lactobacillus* at the genus level in male mice of two groups. (H) The body weight of male mice in the three groups. (I) The expression level of *IL-6* was examined in small intestine tissues from male mice by qRT-PCR. (J-K) The levels of *IL-6* and *TNF-α* in small intestine of male mice were measured by ELISA. (A-C) Significant differences are indicated: Wilcoxon rank sum test. (F-K) Significant differences are indicated: **P* < 0.05, ***P* < 0.01 by Student’s *t*-test between two cohorts.


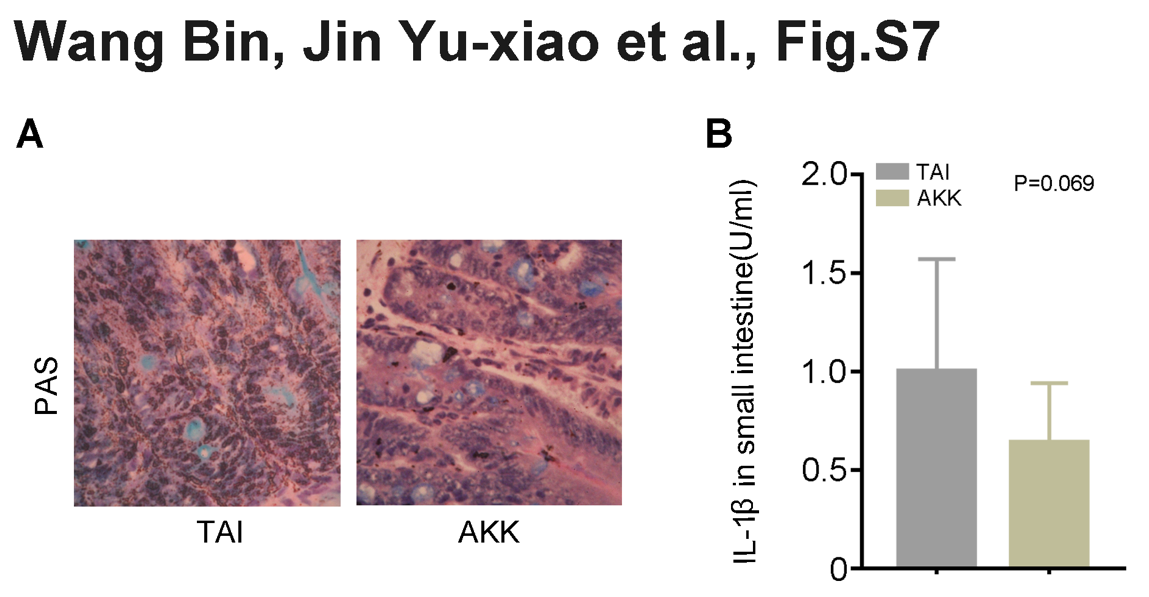


**Fig S7.** *A. muciniphila* supplementation alleviates inflammation levels of gut in irradiation mice. (A) The levels of *IL-1β* in small intestine of male mice were measured by ELISA.


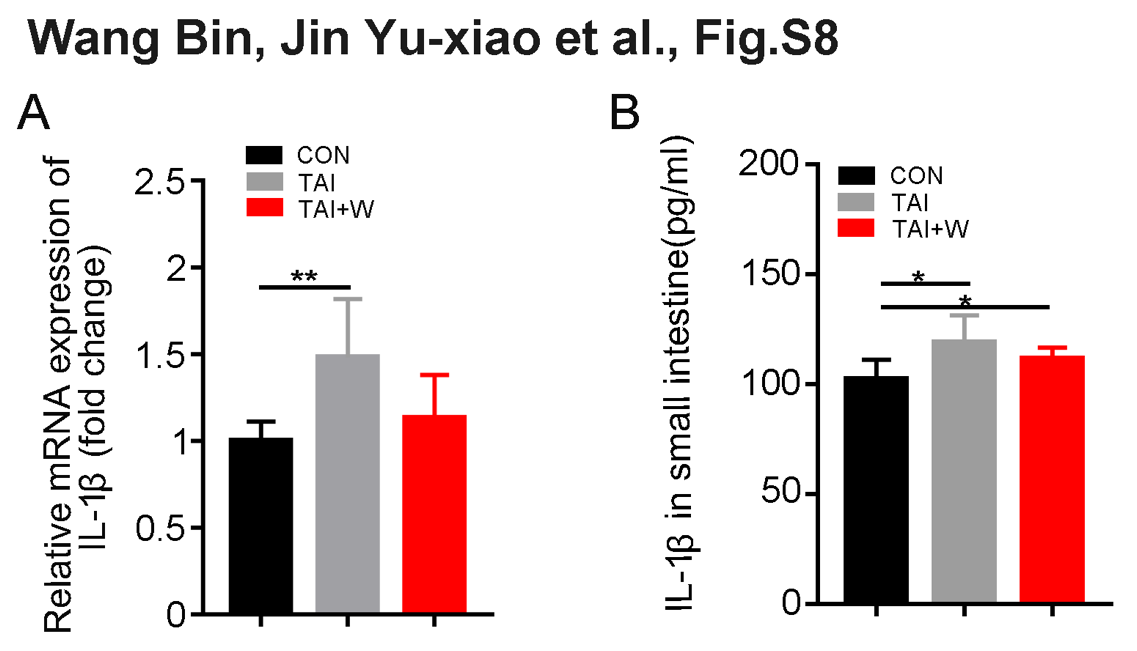


**Fig S8.** Walking works on radiation intestinal injury in female mice. (A) The expression levels of *IL-1β* were examined in small intestine tissues from male mice by qRT-PCR. (B)The levels of *IL-1β* in small intestine of male mice were measured by ELISA. Each experiment was repeated at least three times. The data were presented as mean ± SD. Significant differences are indicated: **P* < 0.05, ***P* < 0.01 by Student’s *t*-test between each two cohorts.
